# Pleural effusion caused by* Trichinella spiralis* infection: two case reports

**DOI:** 10.1186/s12879-023-08047-9

**Published:** 2023-02-06

**Authors:** Zhen-zhen Pan, Miao-juan Zhu, Yu-qiong Rong, Jiong Yang

**Affiliations:** Department of Respiratory and Critical Care Medicine, Zhong-nan Hospital of Wuhan University, 169 Eastlake Road, Wuhan, 430071 China

**Keywords:** *Trichinellosis*, Hydrothorax, Exudate, Enzyme linked immunosorbent assay

## Abstract

**Background:**

*Trichinosis* is a worldwide food-borne zoonotic parasitic disease, which is mainly obtained by ingesting undercooked meat containing infected larvae. The purpose of our article is to introduce and discuss two rare cases of pleural effusion caused by *Trichinella spiralis*.

**Case presentation:**

Here we described two male patients who presented to the respiratory department of our hospital with a massive unilateral pleural effusion, their serum eosinophils were in the normal range, laboratory serological tests revealed that *Trichinella spiralis* IgG antibody was positive. After the oral administration of antiparasitic drugs, the pleural effusion of two patients was completely absorbed.

**Conclusion:**

Both patients were diagnosed with *Trichinosis* complicated with pleural effusion*,* which is very rare in the clinic and easy to be misdiagnosed because of normal eosinophils.

## Background

*Trichinellosis* is a worldwide food-borne zoonotic parasitic disease caused by the infection of all species of *Trichinella *spp, which is mainly acquired by eating undercooked meat containing infected larvae [1]. From 1964 to 2011, more than 600 outbreaks of human *Trichinosis* were recorded in China, which were prevalent in southwest, northeast, and central regions. The outbreaks mainly occurred in Yunnan province, the outbreak time was concentrated in winter and spring, and the majority of people were young adults. The World Organization for Animal Health (OIE) defines this disease as a class B zoonotic disease, and China lists this disease as a class II animal disease, which is required to be detected in pig slaughtering and quarantine. *Trichinella spiralis* infection can cause fever, eyelid edema, muscle pain, eosinophilia, and other symptoms [2]. Severe *Trichinosis* is characterized by cardiovascular, pulmonary, and central nervous system involvement [3]. *Trichinella spiralis* infection causes pleural effusion, which is relatively rare clinically. Because clinicians have little understanding of this disease, it is easy to miss and misdiagnose.

## Case report

### Case 1

**Fig. 1:**
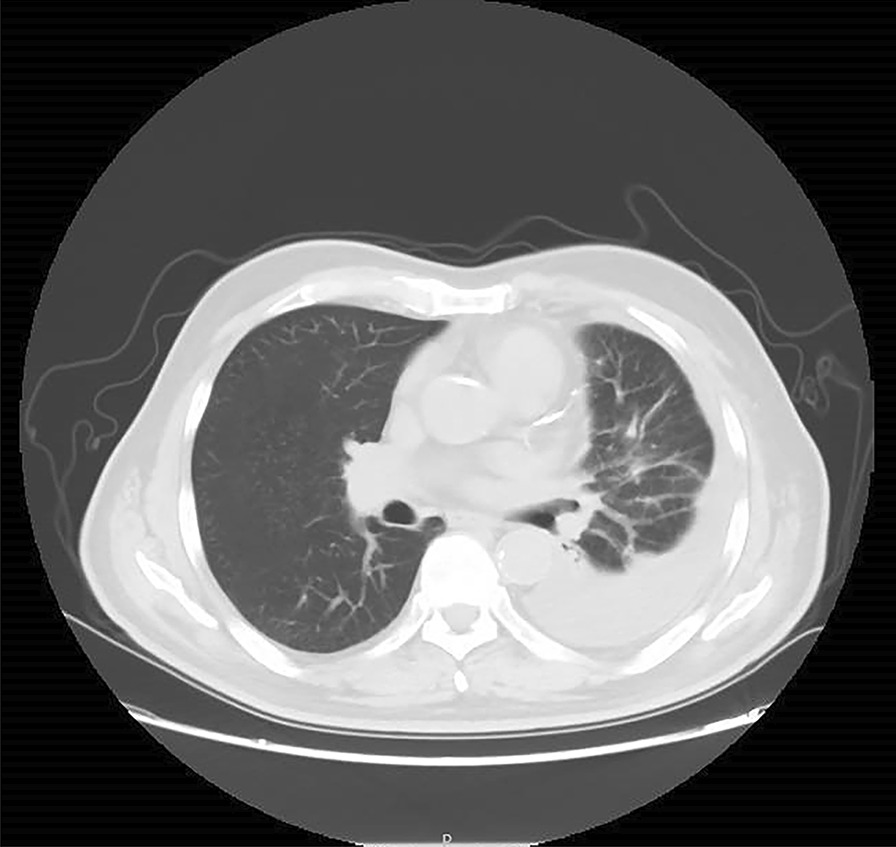
CT of the lung: obvious effusion signs were seen in the left thoracic cavity, patchy consolidation shadows were seen in the left lower lobe with incomplete swelling

**Fig. 2:**
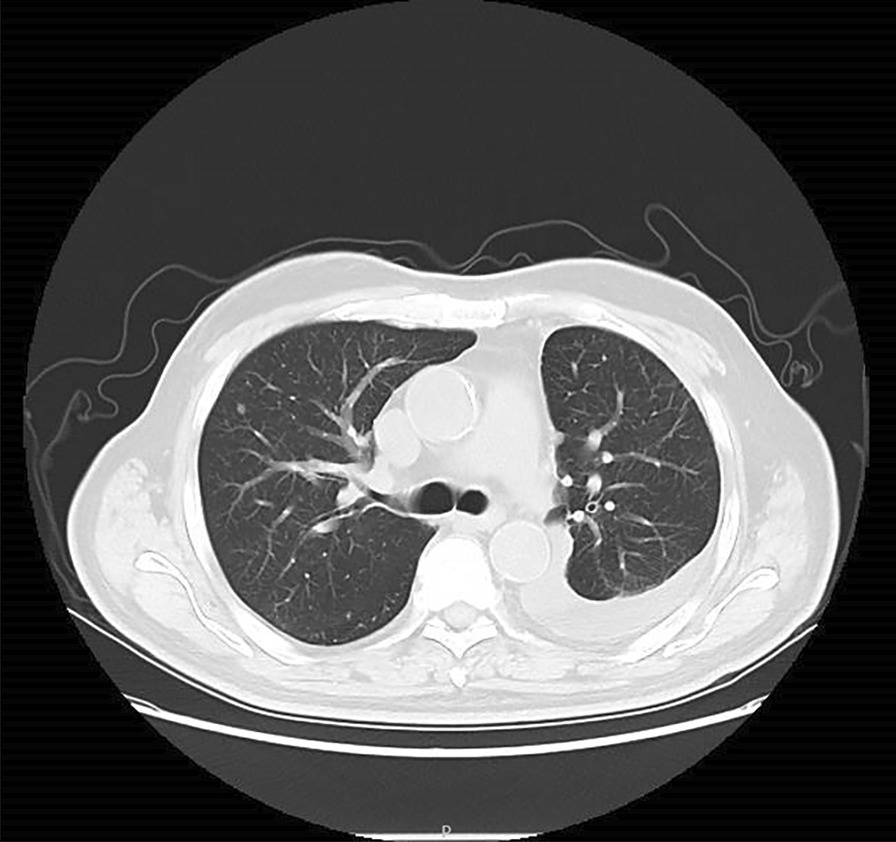
CT of the lung: the right lung is scattered in solid nodules

A 75-year-old man was admitted to the hospital on December 20th, 2020, because his left pleural effusion was found by physical examination for half a month. He had no obvious edema around the eyes and face, and no muscle pain. Previous history of hypertension. Admission physical examination: Temperature: 36.8 ℃, Pulse: 78 times/min, Respiratory Rate: 20 times/min, Blood Pressure: 143/67 mmHg, Low breath sounds in the lower left lung, normal auscultation in the right lung. Lung Computed Tomography (CT) in our hospital on December 18th, 2020: Moderate pleural effusion on the left side with partial distension of the left lung; the right lung is scattered in solid nodules (Figs. 1, 2). After the patient was admitted to the hospital, the absolute value of serum eosinophils was 0.30 × 10^9^/L (the normal range is 0.05–0.50 × 10^9^/L), Serum creatine kinase was 48 u/L (the normal range is 38–174 u/L). Other serum laboratory tests were normal. Serum protein was 55.1 g/L (the normal range is 65–85 g/L), and serum lactate dehydrogenase (LDH) was 141 u/L (the normal range is 125–243 u/L). Routine examination of pleural effusion: Color: red; Transparency: turbid; Coagulation: there are clots; Specific gravity: 1.022; Rivalta test: positive; Number of nucleated cells: 3180/ul; Mononuclear cells (included lymphocytes and monocytes): 98.9%; Multiple nuclear cells (included neutrophils and eosinophils): 1.1%. Biochemical examination of pleural effusion: Glucose: 5.52 mmol/L (the normal range is 3.9–6.1 mmol/L); Protein: 36.5 g/L (the normal range is 20–40 g/L); Albumin: 22.2 g/L (the normal range is 20–40 g/L); Chlorine: 111.3 mmol/L (the normal range is 110-130 mmol/L); Pleural LDH: 220 U/L (the normal range is 230–460 u/L); Adenosine deaminase (ADA):9U/L(the normal range is 0–18 u/L). No obvious abnormality was found in urological and cardiac ultrasound. Chest ultrasound suggested massive effusion in the left pleural cavity (the maximum anteroposterior diameter is about 7.6 cm). Positron emission tomography/computed tomography (PET/CT) showed: No signs of muscle involvement; no obvious signs of malignant tumor lesions were found at the detection site.

The patient received an anti-infective treatment (cefotaxime sodium and sulbactam) for 1 week and then the pleural effusion had no significant decrease. Cytological report of pleural effusion showed that there were a few lymphocytes and mesothelial cells, and no obvious atypical epithelial cells. On the 11th day of admission, he was given diagnostic anti-*Tuberculosis* treatment for 25 days (rifampicin 450 mg quaque die (QD) + isoniazid 0.3gQD + moxifloxacin 0.4gQD). Pleural ultrasonography showed no obvious pleural fluid absorption, so he stopped using anti-*Tuberculosis* drugs. We questioned closely the patient's past history, He complained that he ate undercooked pork sausages sold in a small shop a month and a half ago, and after that, symptoms of diarrhea occurred (about 4 days). At this time, we considered whether there was parasitic infection. Therefore, the patient's serum was sent to Wuhan Tongji hospital, and the detection of parasite antibody IgG indicated *Trichinella spiralis* (+) by enzyme-linked immunosorbent assay (ELISA), and the diagnosis was *Trichinosis*. Albendazole tablets were given 800 mg/d for 1 week. Two weeks later, a chest ultrasound showed that the pleural effusion disappeared.

### Case 2

**Fig. 3:**
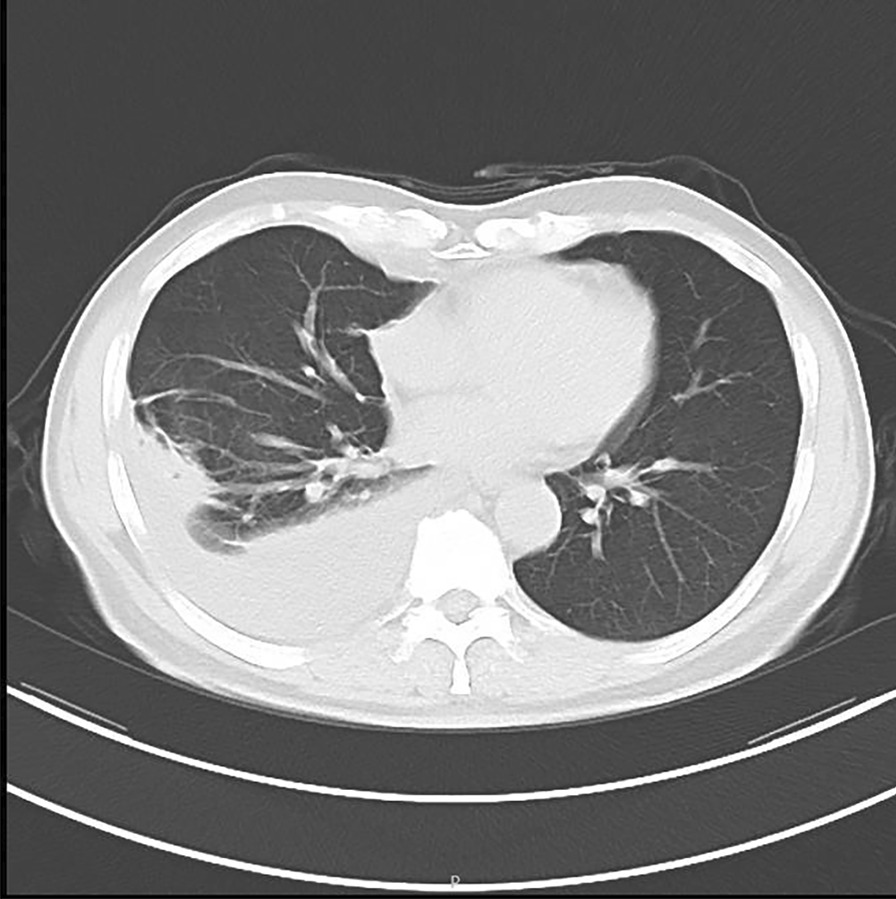
CT of the lung: patchy shadow can be seen in the lower lobe of the right lung, and effusion signs can be seen in the right chest cavity with hypodynamia of the lower lobe of the right lung

**Fig. 4:**
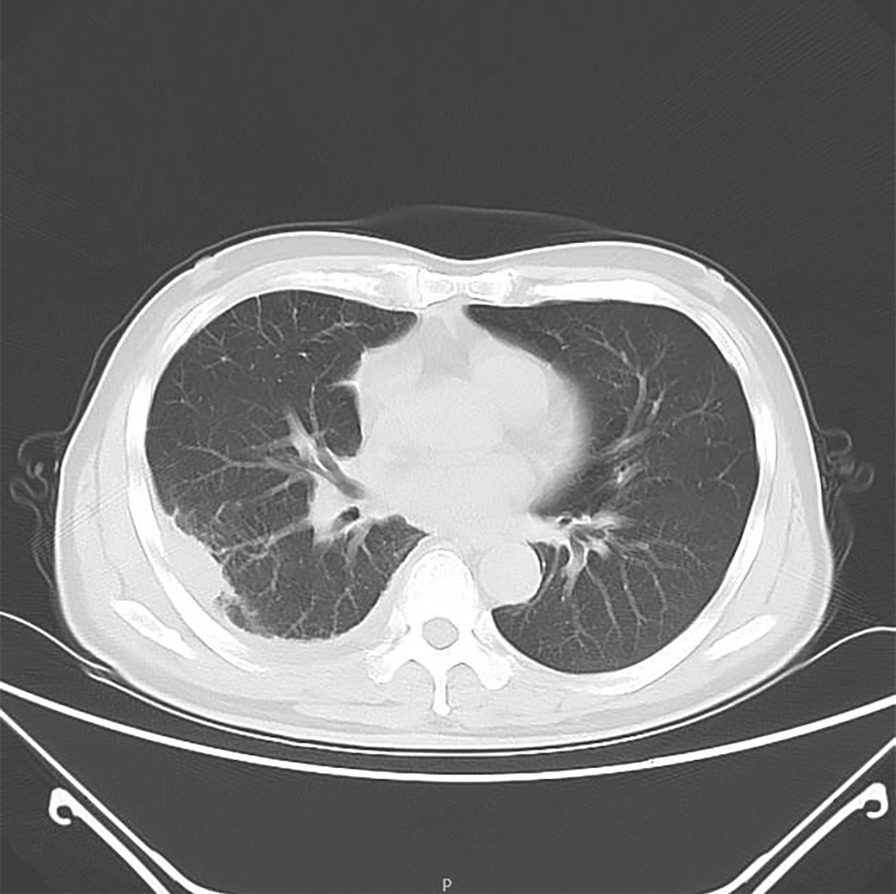
CT of the lung: after treatment, the right pleural effusion was significantly absorbed and the lung tissue was restretched

A 59-year-old male was admitted to the hospital on November 24th, 2021 because of a cough for 1 week. The patient's cough was a mainly dry cough, accompanied by chest wall pain and wheezing at night. The symptoms worsened two days ago, accompanied by white phlegm and hoarseness, but no fever, so he came to our hospital for further treatment. Past physical fitness. Physical examination on admission: Temperature: 36.5 ℃, Pulse: 113 times/min, Respiratory Rate: 19 times/min, Blood Pressure: 119/79 mmHg, SpO_2_: 98%. A little moist rale can be heard in the upper right lung, and the left lung was normal, without wheezing or bronchospasm on lung auscultation. After the patient was admitted to the hospital, the absolute value of serum eosinophils was 0.2 × 10^9^/L (the normal range is 0.05–0.5 × 10^9^/L), other serum laboratory tests were normal. Electrocardiogram and purified *Tuberculin* pure protein derivative (PPD) tests were normal. Serum protein was 61.3 g/L. Routine examination of pleural effusion: color: light yellow; Transparency: slightly turbid; Coagulation: there are clots; Specific gravity: small quantity; Rivalta test: positive; Number of nucleated cells: 2488/ul; Mononuclear cells (included lymphocytes and monocytes): 86.7%; Multiple nuclear cells (included neutrophils and eosinophils): 13.3%. Biochemical examination of pleural effusion: Glucose: 7.38 mmol/L; Protein: 43.4 g/L; Albumin: 28.0 g/L; Chlorine: 108.9 mmol/L; Pleural LDH: 636 U/L; ADA:2U/L. Cytological examination of pleural effusion showed that a large number of inflammatory cells (mainly lymphocytes and neutrophils) and a few tissue cells were observed under a microscope. CT of the lung: Inflammation of the right lower lobe of the lung; Right pleural effusion with right lower lobe atelectasis (Fig. 3). Chest ultrasound suggested a massive pleural effusion on the right side (maximum anteroposterior diameter is about 10.7 cm). On the 8th day after admission, the patient's serum was sent to Wuhan Tongji hospital, and the detection of parasite antibody IgG indicated *Trichinella spiralis* (+) by ELISA, and the diagnosis was *Trichinosis*. After oral treatment with mebendazole tablets 300 mg/d for 1 week, his cough symptoms were completely relieved, one month later, the reexamination of lung CT showed that the right pleural effusion was absorbed (Fig. 4).

## Discussion

*Trichinella spiralis* belongs to nematodes. *Trichinella spiralis* can infect pigs, dogs, cattle, cats, wolves, weasels, minks, foxes, whales, and others. Human infection with *Trichinella* is mainly due to eating raw or semi-raw animal meat (pork, wild boar, dog meat, etc.). *Trichinosis* patients themselves are not contagious, and their typical clinical manifestations after infection are high fever, periorbital and facial edema, muscle pain, subconjunctival hemorrhage, an allergic rash, and crescent or linear hemorrhage under fingers or toenails. Severe patients may have complications such as myocarditis, encephalitis, or pneumonia [4]. According to statistics from the zoonotic disease surveillance conducted by the European Union (MS) in 2017, half of the member states reported zero cases, and these countries have never reported any cases of *Trichinosis* [5]. In China, *trichinellosis* is mainly prevalent in the southwest, northeast, and central regions, and the outbreak mainly occurred in Yunnan Province.

The diagnosis of human *Trichinellosis* is mainly based on clinical symptoms and serological tests, such as ELISA, indirect immunofluorescence assay (IFA), enzyme immunohistochemistry technique (EIH), and Western-Blotting (WB), The International Commission on Trichinosis recommends ELISA, which detects anti-trichinella IgG in patients' serum by excretory secretion (E/S) antigen of *Trichinella* spiralis larvae. The sensitivity and specificity of total IgG were 93.6% and 94.3%, respectively [6]. The limitations of this method are that it has a high false-negative rate in the early stage of infection (1–3 months) and cannot distinguish between acute and previous infection [7]. Detection of circulating antigens (CAg), such as anti-immunoelectrophoresis, immunoradiometric assay, direct ELISA, and sandwich ELISA, is an effective method to distinguish between acute and previous infections. CAg is an excretory or secretory antigen produced by live insects, which can directly enter the peripheral blood circulation and can be used for early diagnosis and curative effect evaluation of *Trichinosis*. However, the detection rate of circulating antigens in serum is usually only 30–50% [8]. Therefore, it is not recommended for the diagnosis of *Trichinosis*. The discovery of *Trichinella* larvae cysts from muscle biopsy is the gold standard for the diagnosis of *Trichinosis*, but due to its invasiveness, the positive rate is only about 50%, and early infection cannot be detected, so it is not recommended [9]. The European Center for Disease Control has classified human *Trichinosis* into the clinical, laboratory, and epidemiological cases, of which laboratory cases refer to *Trichinosis* larvae found by muscle biopsy or seropositive. In this paper, the patient had a history of eating pork sausage, and the serological test was positive. After being treated with insect repellent (albendazole or mebendazole), pleural effusion was absorbed, so it was diagnosed as *Trichinosis*. The treatment plan for *Trichinosis* includes antiparasitic drugs (mebendazole or albendazole) and glucocorticoids [10]. Our patients were not given glucocorticoids during treatment. Albendazole is currently the preferred drug for the treatment of *Trichinosis* in China, the dosage is 20–30 mg/(kg·d), divided into two oral courses for 5–7 days [10]. Most patients have a good prognosis and recover within 1–2 months.

We summarized some cases of pleural effusion caused by a parasitic infection in Table 1 [11–20], the eosinophils of patients with parasitic pleurisy can be in the normal range, and the diagnosis of parasitic pleurisy mainly relies on ELISA of antibodies directed, such as *Paragonimiasis, Toxocariasis,* and* Hydatidosis* [21, 22]. The pleural effusion in the two patients in this paper was unilateral, and the pleural effusion was a lymphocyte-dominant exudate. The level of ADA was low, and it was difficult to diagnose *Tuberculous pleuritis* [23]. The tumor, rheumatism immunity, and other related tests were negative, and there was no significant increase in eosinophils in blood and pleural effusion, so we didn't consider parasitic infection when two patients were admitted to the hospital. The patient of case 1, after further questioning the medical history, he complained that he had recently eaten undercooked pork sausages sold in small shops. Considering that *Paragonimiasis* pleural effusion caused by eating crayfish was found in the Wuhan area. The clinical manifestations and pleural effusion properties of the two patients in this article are very similar. Therefore, we added serum parasite antibody tests. For case 1, it was speculated that the onset stage of the disease was *Trichinosis* when the patient ate undercooked pork sausage before 1.5 months, and the acute attack showed gastrointestinal symptoms (diarrhea). Then, pleural effusion was found in the physical examination before a month, which was considered as the late stage of *Trichinosis* and complications of pleural effusion. Studies have proved that respiratory complications are mostly in the late stage of the disease, that is, between the 3rd and 7th week of infection, bacterial pneumonia, pleurisy, and lung infarction may occur [24]. Therefore, the final diagnosis was *Trichinosis* complicated with pleural effusion in the late stage.

**Table 1:** Summary of pleural effusion caused by parasite infection

References	Patient’s age/sex	Country	Diagnosis	Disease history	Symptoms	Abnormal inspection result	Confirmed conditions	Treatment	Outcome
Soukup et al. [11]	28/female	Krajska zdravotni	Toxocariasis/helminthozoonosis	History of substance abuse and chronic type C hepatitis	After the second procedure, which was a vertebral body replacement via thoracotomy, the patient developed a pathologic pleural effusion with asymptomatic	A relatively high number of immunocompetent cells (6 830/1 µL), with a slight predominance of monocytes and macrophages (about 40%) and a smaller number of neutrophils (about 30%), lymphocytes (about 15%), and eosinophils (about 15%). No increase in the number of eosinophilic granulocytes and no leukocytosis were found in the blood tests	Enzyme-linked immunosorbent assay (ELISA) and Western blot were negative, microscopic evaluation was positive	Albendazole 400 mgQD/7d	No permanent sequelae of the infection
Fan et al. [12]	65/female	Qinghai Province in China	Cutaneous myiasis with eosinophilic pleural effusion	Occasional dietary habit of eating raw meat	Recurrent cough, occasional hemoptysis, and right chest pain	A left hydropneumothorax with partial compressive atelectasis and patchy consolidation on the right lung. Laboratory data revealed peripheral blood eosinophilia of 37.2%, with a white blood cell count of 10.4 × 109/L. Serum immunoglobulin E levels were elevated (1650 unit/mL)	Tender nodules and worm-like live organisms were observed in her upper arms and shoulders. cysticercosis IgG(+)	Albendazole (400 mg/d) for 3 days	Telephonic follow-up 1 month later indicated that the blood eosinophilia and pleural effusion were resolved
Park et al. [13]	21/female	Korean	Toxocariasis	None	Epigastric pain, vomiting, headache, and dizziness	Right pleural effusion, pericardial effusion, and focal ascites in the pelvic cavity. Laboratory tests revealed an elevation of eosinophils (40%) and cardiac enzymes (creatinine kinase-MB 27.6 ng/mL, high-sensitive cardiac troponin T 1.21 ng/mL). The transthoracic echocardiogram showed left ventricular systolic dysfunction (ejection fraction 44%) and moderate pericardial effusion	The serologic test for parasites was positive for Toxocara and Sparganum	A combination therapy of albendazole (400 mgBID/2 weeks), praziquantel (600 mgTID/1 day), and corticosteroid (60 mgQD/6 days)	At Outpatient Clinic follow-ups and observations over the next 2 years there were no abnormal findings, including pericardial effusion or eosinophilia
Savu et al. [14]	43/male	Romania	Hydatidosis caused by the Echinococcus larvae	He was a heavy smoker, occasional consumer of ethanol as well as working with livestock as a shepherd	Moderate dyspnea, chest pain and weight loss	Multiple cystic formations of various sizes and liquid density within the pleural fluid	Elevated eosinophil count, Surgery was performed by right lateral thoracotomy and consisted of removal of the hydatid fluid and cysts found in the pleura. IGG-specific ELISA tests(+)	Albendazole 15 mg/kg/day for 6 days before surgery, Albendazole treatment for 1 year with 15 mg/kg/day	Follow-up showed no signs of recurrence with a normal chest X-ray and an improved lung volume function at one month, 6 months and 1 year
Aggarwal et al. [15]	30/male	Uttar Pradesh, India	Microfilaria/ Filariasis	None	Low-grade, intermittent fever for two years, right-sided chest pain and weight loss	Chest radiography showed a right-sided pleural effusion with normal parenchymal attenuation	Fluid cytology showed degenerated lymphocytes along with a few microfilaria, conforming to the morphology of Wuchereria Bancrofti	Diethylcarbamazine 300 mg daily in divided doses	Chest radiography repeated 6 weeks later showed complete clearance of the effusion
Tourne M et al. [16]	39	Paris	Cystic echinococcosis, or hydatidosis	None	Chest pain associated with sweating and chills	Thoracic computed tomography shows two large cystic opacities with endocystic flaky images, including one ruptured in the pleura with right pleural effusion	Positive hydatidosis serology, and surgery	Treatment by albendazole	full-recovery
Hämäläinen et al. [17]	8/child	eastern Finland	Cystic echinococcosis (CE) or hydatidosis	None	Abdominal pain with a vigorous generalised urticarial rash, fever (38.5 °C) and a persistent cough	The ultrasound showed a considerable avocado-sized hollow (13.5 × 9 cm) with multiple lobulation	C-reactive protein (58 mg/L; norm: < 3 mg/L), Elevation of the serum eosinophil leucocytes (4.7 × 109/L; norm: 0.1–0.4 × 109/L). the direct microscopic examination of calcofluor white-stained fragments of cyst wall and cyst content showed plenty of hooks and protoscolices, surgery	Albendazole treatment (10–15 mg/kg/day divided in two doses) was continued postoperatively for a total of 3 months	Control specimens taken 1 month after deworming were PCR-negative
Vallentin et al. [18]	5/female	Romania	Toxocariasis	None	Asymptomatic	The chest X-ray showed an abundant left pleural effusion and a lower lobar atelectasis	A marked hypereosinophilia (2.1 × 10^9/L, with a maximum few days later of 7.2 × 10^9/L), Serological tests were positive only for Toxocara canis (by Western Blot and Elisa techniques)	Treatment with albendazole 15 mg/kg/d was initiated for 15 days	The control of the chest X-ray 6 weeks later was normal
Oh et al. [19]	45/male	Korea	Sparganosis	Occasional frog and snake consumption from the age of 25 years	Left lower chest pain, a total body skin rash, cough, sputum production, abdominal discomfort, and a febrile sense for 1 week	Localized pleural effusion in the left lower lobe, peripheral blood eosinophilia and eosinophilic pleural effusion were present	Percutaneous catheter drainage was performed, which revealed long worm-shaped material that was identified as a sparganum by DNA sequencing	Praziquantel	At follow-up 1 month later, he presented with normal peripheral eosinophilia and a complete clinical recovery
Hernández et al. [20]	34/female	Colombia	Chagas disease, caused by infection with the parasite Trypanosoma cruzi	With an HIV infection, not receiving antiretroviral treatment,	Chest pain associated with dyspnea, weight loss, asthenia, adynamia, and hyporexia	Extensive pleural effusion in the right hemithorax, Brain tomography showed a bifrontal hypodense left lesion and cerebral edema,	Flagellated parasites consistent with trypomastigotes were observed in both fluids (pleural and CSF)	Therapy with nifurtimox 8 mg/kg/day was administered over the course of 4 days following diagnosis	The patient died 1 day after therapy ceased

## Conclusion

*Trichinosis* causes unilateral pleural effusion, which is extremely rare in clinical practice and has not been reported yet. The purpose of this paper is to remind clinicians that parasitic diseases should be included in the differential diagnosis of patients with unexplained pleural effusion, even if their serum eosinophils are within the normal range.

## Data Availability

All data generated or analysed during this study are included in this published article.

## Abbreviations

CT: Computed Tomography
G test: 1,3-Beta-D glucan test
GM test: Galactomannan test
LDH: Lactate dehydrogenase
ADA: Adenosine deaminase
PET/CT: Positron emission tomography/computed tomography
QD: Quaque die
PPD: Tuberculin pure protein derivative
ELISA: Enzyme-linked immunosorbent assay
CAg: Circulating antigens

## References

[1] Capo V, Despommier DD (1996). Clinical aspects of infection with *Trichinella* spp. Clin Microbiol Rev.

[2] Wilson NO, Hall RL, Montgomery SP, Jones JL (2015). Trichinellosis surveillance–United States, 2008–2012. Morb Mortal Wkly Rep Surveill Summ.

[3] Taratuto AL, Venturiello SM (1997). Trichinosis. Brain Pathol.

[4] Diaz JH, Warren RJ, Oster MJ (2020). The disease ecology, epidemiology, clinical manifestations, and management of trichinellosis linked to consumption of wild animal meat. Wilderness Environ Med.

[5] European Food Safety Authority and European Centre for Disease Prevention and Control (EFSA and ECDC (2018). The European Union summary report on trends and sources of zoonoses, zoonotic agents and food-borne outbreaks in 2017. EFSA J Eur Food Saf Auth.

[6] Kahsay R, Gómez-Morales MA, Rivera HN, McAuliffe I, Pozio E, Handali S (2021). A Bead-based assay for the detection of antibodies against *Trichinella* spp. infection in humans. Am J Trop Med Hyg.

[7] Wang ZQ, Fu GY, Jing FJ, Jin J, Ren HJ, Jiang P, Cui J (2012). Detection of *Trichinella spiralis* circulating antigens in serum of experimentally infected mice by an IgY-mAb sandwich ELISA. Foodborne Pathog Dis.

[8] Nishiyama T, Araki T, Mizuno N, Wada T, Ide T, Yamaguchi T (1992). Detection of circulating antigens in human trichinellosis. Trans R Soc Trop Med Hyg.

[9] Thawornkuno C, Nogrado K, Adisakwattana P, Thiangtrongjit T, Reamtong O (2022). Identification and profiling of *Trichinella spiralis* circulating antigens and proteins in sera of mice with trichinellosis. PLoS ONE.

[10] Dupouy-Camet J, Kociecka W, Bruschi F, Bolas-Fernandez F, Pozio E (2002). Opinion on the diagnosis and treatment of human trichinellosis. Expert Opin Pharmacother.

[11] Soukup J, Cerny J, Cegan M, Kelbich P, Novotny T (2021). Toxocariasis as a rare parasitic complication of a transthoracic spine surgery procedure. Medicina.

[12] Fan T, Zhang Y, Lv Y, Chang J, Bauer BA, Yang J, Wang CW (2021). Cutaneous myiasis with eosinophilic pleural effusion: a case report. World J Clin Cases.

[13] Park SJ, Jang CW, Kim YK, Seo YH, Kim KH, Kwon TG, Bae JH (2021). Toxocariasis-associated acute perimyocarditis with cardiogenic shock: a case report. Am J Case Rep.

[14] Savu C, Melinte A, Grigorie V, Iliescu L, Diaconu C, Dimitriu M, Socea B, Stiru O, Varlas V, Savu C (2020). Primary pleural hydatidosis-a rare occurrence: a case report and literature review. Medicina.

[15] Aggarwal P, Subramanian S, Saini V, Aggarwal D (2021). Filariasis presenting as isolated pleural effusion: a case report and mini review. Trop Doct.

[16] Tourne M, Dupin C, Mordant P, Neuville M, Taillé C, Danel C (2019). Autochthonous hydatid cyst of the lung. Ann Pathol.

[17] Hämäläinen S, Kantele A, Arvonen M, Hakala T, Karhukorpi J, Heikkinen J, Berg E, Vanamo K, Tyrväinen E, Heiskanen-Kosma T (2015). An autochthonous case of cystic echinococcosis in Finland, 2015. Eur Commun Dis Bull.

[18] Vallentin B, Carsin A, Dubus JC (2015). Toxocariasis: an unusual cause of pleural effusion. Pediatr Pulmonol.

[19] Oh Y, Kim JT, Kim MK, Chang YJ, Eom K, Park JG, Lee KM, Choe KH, An JY (2014). *Eosinophilic pleuritis* due to sparganum: a case report. Korean J Parasitol.

[20] Hernandez C, Cucunuba Z, Parra E, Toro G, Zambrano P, Ramírez JD (2014). Chagas disease (*Trypanosoma cruzi*) and HIV co-infection in Colombia. Int J Infect Dis.

[21] Wang J, Luo W, Shen P, He J, Zeng Y (2019). Retrospective study of pleural parasitic infestations: a practical diagnostic approach. BMC Infect Dis.

[22] Al-Tawfiq JA, Kim H, Memish ZA (2022). Parasitic lung diseases. Eur Respir Rev.

[23] Ferreiro L, San José E, Valdés L (2014). Tuberculous pleural effusion. Arch Bronconeumol.

[24] Kociecka W (2000). Trichinellosis: human disease, diagnosis and treatment. Vet Parasitol.

